# New antidiabetic therapy and HFpEF: light at the end of tunnel?

**DOI:** 10.1007/s10741-021-10106-9

**Published:** 2021-04-11

**Authors:** Marijana Tadic, Carla Sala, Sahrai Saeed, Guido Grassi, Giuseppe Mancia, Wolfang Rottbauer, Cesare Cuspidi

**Affiliations:** 1 Klinik Für Innere Medizin II, Universitätsklinikum Ulm, Albert-Einstein Allee 23, 89081 Ulm, Germany; 2 Department of Clinical Sciences and Community Health, University of Milano and Fondazione Ospedale Maggiore IRCCS Policlinico Di Milano, Milan, Italy; 3 Department of Heart Disease, Haukeland University Hospital, Bergen, Norway; 4 Clinica Medica, University of Milan-Bicocca, Milan, Italy; 5 Milano and Policlinico Di Monza, University of Milano-Bicocca, Monza, Italy; 6 Istituto Auxologico Italiano, IRCCS, Milan, Italy

**Keywords:** SGLT2 inhibitors, GLP-1 receptor agonists, DPP-4 inhibitors, Left ventricular, Diastolic function, Heart failure

## Abstract

New antidiabetic therapy that includes sodium-glucose co-transporter 2 (SGLT2) inhibitors, glucagon-like peptide-1 receptor (GLP-1R) agonists, and dipeptidyl peptidase 4 (DPP-4) inhibitors showed significant benefit on cardiovascular outcomes in patients with and without type 2 diabetes mellitus, and this was particularly confirmed for SGLT2 inhibitors in subjects with heart failure (HF) with reduced ejection fraction (HFrEF). Their role on patients with HF with preserved ejection fraction (HFpEF) is still not elucidated, but encouraging results coming from the clinical studies indicate their beneficial role. The role of GLP-1R agonists and particularly DPP-4 inhibitors is less clear and debatable. Findings from the meta-analyses are sending positive message about the use of GLP-1R agonists in HFrEF therapy and revealed the improvement of left ventricular (LV) diastolic function in HFpEF. Nevertheless, the relevant medical societies still consider their effect as neutral or insufficiently investigated in HF patients. The impact of DPP-4 inhibitors in HF is the most controversial due to conflicting data that range from negative impact and increased risk of hospitalization due to HF, throughout neutral effect, to beneficial influence on LV diastolic dysfunction. However, this is a very heterogeneous group of medications and some professional societies made clear discrepancy between saxagliptin that might increase risk of HF hospitalization and those DPP-4 inhibitors that have no effect on hospitalization. The aim of this review is to summarize current clinical evidence about the effect of new antidiabetic medications on LV diastolic function and their potential benefits in HFpEF patients.

## Introduction

Heart failure with preserved ejection fraction (HFpEF) makes almost the half of all patients with heart failure (HF) [1]. The existing trend of increased HFpEF prevalence is the consequence of elevated incidence of hypertension, diabetes, and obesity, as well as normal aging of population [1]. Significant improvement in the cardiovascular imaging techniques and particularly echocardiography and magnetic resonance imaging has contributed to early detection and more precise evaluation of left ventricular (LV) diastolic dysfunction and increased number of diagnosed HFpEF patients.

There are no guidelines for specific treatment of HFpEF. The same medications used in the large therapeutic armamentarium in patients with heart failure with reduced ejection fraction (HFrEF) are used in HFpEF. Nevertheless, the significant improvement was made in the treatment of HFrEF in the last decade and some new medications with new mechanisms of actions were approved for clinical usage. One of the most important is the angiotensin receptor II blocker-neprilysin inhibitor (ARNI), sacubitril-valsartan that has significantly changed the practice in HFrEF treatment and became an established part of therapy in these patients [2]. However, the impact of sacubitril-valsartan in HFpEF is still not fully understood. Results from studies are still controversial, but latest data showed beneficial effect in some HFpEF patients with lower range of left ventricular ejection fraction (LVEF) and women [3], which is why the Federal Drug Agency recently approved sacubitril-valsartan in these patients [4].

More recently new antidiabetic medications showed significant improvement in patients with HFrEF regardless of the presence of DM [5–7], but data in HFpEF are still scarce until ongoing trials do not provide evidence [8]. Sodium-glucose co-transporter 2 (SGLT2) inhibitors, glucagon-like peptide-1 receptor (GLP-1R) agonists, and dipeptidyl peptidase 4 (DPP-4) inhibitors showed significant benefit on cardiovascular outcomes in both patients with and without type 2 diabetes mellitus (DM) [2].

The aim of this review is to summarize current clinical knowledge about the effect of new antidiabetic medications on LV diastolic function and potential clinical benefits in HFpEF patients.

## The effects of SGLT2 inhibitors on cardiovascular system

Preclinical and clinical studies showed significant positive pleotropic effects on kidneys, liver, pancreas, blood plasma, blood vessels, and adipose tissue that result with decreased preload and afterload, reduced fibrosis, oxygen demand, and LV hypertrophy, as well as decreased afterload [9]. The effect of SGLT2 inhibitors is achieved through SGLT2, a sodium-glucose co-transporter located on the apical membrane of the renal proximal convoluted tubules. It is responsible for more than 90% of glucose reabsorption in the kidney, whereas the rest is achieved by SGLT1 in the descending arm of the loop of Henle [9]. The difference in glucose concentration between cytoplasm and plasma enables the passive glucose transport through the basolateral membrane. The glicosuria resulting from SGLT2 inhibition is proportional to the level of blood glucose. SGLT2 inhibitors show a modest efficacy in lowering plasma glucose levels, reducing HbA1c by approximately 0.5–1%.

The diuretic and natriuretic effects of SGLT2 inhibitors induce depletion in plasma volume, which is the most important to prevent fluid retention and HF exacerbation. At difference from traditional diuretics, these medications have been shown to reduce not only intravascular volume but also interstitial fluid; this mechanism may exert adverse effects in HF patients with reduced intravascular volume and induce diuretic resistance.

The diuretic effect of SGLT2 inhibitors is associated with reduced preload and LV filling pressure, myocardial stretching, and myocardial interstitial fibrosis. Furthermore, the reduction in extracellular fluid volume is associated with decrease in blood pressure [10, 11], which is even more amplified with the reduction in body mass induced by SGLT2 inhibitors [11]. This weight loss is related with increased glucose excretion in the urine and loss of extracellular fluid. Böhm et al. reported that SGLT2 inhibitor (empagliflozin) reduced risk of heart failure, as well as cardiovascular and renal outcomes independently of mean systolic blood pressure during the trial [12]. These results suggest a BP-independent effect of empagliflozin on cardiovascular and heart failure outcomes.

Studies also showed that SGLT2 inhibitors reduced albuminuria, the urinary albumin to creatinine ratio, and slowed the progression from microalbuminuria to macroalbuminuria [13]. Investigations also reported improved endothelial function, reduced oxidative stress and inflammation, and decreased aortic stiffness in patients treated with SGLT2 inhibitors [14]. These mechanisms are associated with reduction in central blood pressure, pulse pressure, and forward wave amplitude [15], which can be helpful in patients with HF. Recent investigations revealed sympathetic nervous system reduction in patients treated with SGLT2 inhibitors [16], which might partly explain beneficial effect in patients with HF.

### SGLT2 inhibitors and LV diastolic function

Studies that involved patients with DM showed significant improvement in LV diastolic function I patients treated with SGLT2 inhibitors [17–23] (Table 1). Meta-analysis that investigated effects of antidiabetic drug on LV function showed that SGLT-2 inhibitors were more significantly associated with improved LV end-diastolic diameter and E/e′ [17]. There was no significant difference in mean change in the treatment effect of *e*′ and *E*/*A* between any of the 6 drugs and placebo or in pairwise comparisons between any two of the 6 drugs (SGLT2 inhibitors, DPP4 inhibitors, GLP1 agonists, metformin, sulfonylurea, and thiazolidinediones [17].

**Table 1:** SGLT2 inhibitors, left ventricular diastolic function and HFpEF

Reference	Sample size	Medication	Follow-up period	Main findings
Verma et al. [18]	10 DM patients with normal LVEF	Empagliflozin	3 months	Significant reduction in LV mass index and improved LV diastolic function (↑e′)
Cohen et al. [19]	20 DM patients with normal LVEF	Empagliflozin	6 months	Reduction in LVEDV without differences in measures of LV mass, LVEF and cardiac fibrosis
Rau et al. [20]	42 DM patients with preserved LVEF (≈50%)	Empagliflozin	3 months	Significantly improved LV diastolic function by reduction of E/e′, but did not change LV longitudinal strain
Lan et al. [21]	44 DM patients after ACS	Empagliflozin	6 months	Reduction in LV mass index, LA volume index and increase in E/e′, withount change in LV longitudinal strain and LVEF
Eickhoff et al. [22]	36 DM with normal LVEF	Dapagliflozin	12 weeks	Dapagliflozin did not have effect on LVEF, LV mass index and E/e′. The composite score showed LV diastolic function improvement of 19.8%
Zhang et al. [17]	4790 DM patients (meta-analysis)	Dapagliflozin Empagliflozin Tofogliflozin	-	SGLT-2 inhibitors are more significantly related with improved LVEDD and E/e′
Hwang et al. [23]	202 DM patients with HFpEF, HFrEF and without HF	Dapagliflozin Empagliflozin Impragliflozin	13 months	Significant decrease in LVEDD, improvement in LVEF, reduction in LV mass index, and E/e′ in HF patients. The improvements were more prominent in HF patients than those without HF, and in HFrEF patients than HFpEF patients
Shim et al. [24]	60 DM patients with LV diastolic dysfunction	Dapagliflozin	24 weeks	Dapagliflozin did not significantly affect resting e′ velocity, E/e′, LV mass index, and left atrial volume index, but it significantly improved E/e′ during exercise, which reduced symptoms during effort
Matsutani et al. [25]	38 DM patients with normal LVEF	Canagliflozin	3 months	LV diastolic function (E/e′) was significantly improved after canagliflozin usage
Otagaki et al. [26]	26 DM patients with normal LVEF	Tofogliflozin	6 months	Significantly improved LVEF and increased E/e′
Soga et al. [27]	58 patients with stable HFrEF	Dapagliflozin	6 months	E/e′ significantly decreased, as well as LA volume index and LV mass index significantly decreased
Tanaka et al. [28]	53 DM patients with stable HFpEF	Dapagliflozin	6 months	Dapagliflozin was found to be associated with improvement of LV longitudinal myocardial strain, which induced further improvement of LV diastolic function of DM patients with stable HFpEF
Sezai et al. [29]	35 DM patients with stable HFpEF	Canagliflozin	12 months	Improved LV diastolic function

*ACS* acute coronary syndrome *DM* diabetes mellitus *HF* heart failure *HFpEF* heart failure with preserved ejection fraction *HFrEF* heart failure with reduced ejection fraction *LA* left atrium *LV* left ventricle *LVEF* left ventricular ejection fraction *SGLT2* sodium-glucose co-transporter 2 inhibitors

Empagliflozin given in subjects with DM and history of cardiovascular (CV) disease showed reduction in LV mass index and LV end-diastolic volume, as well as better parameters of LV diastolic function after 3 or 6 months of therapy [18, 19]. More recent data from the EMPA-REG OUTCOME trial revealed that empagliflozin treatment of patients with DM had no significant effect on hemodynamic parameters during 3 months of therapy, but it induced rapid and sustained improvement of LV diastolic function (E/e′) [20]. Therapy with SGLT2 inhibitors proscribed after acute myocardial infarction was not associated with better LVEF or LV longitudinal strain, but it was related with favorable changes in diastolic function parameters [21]. Other investigation reported only minor effect of empagliflozin on LV diastolic function in DM patients with albuminuria and preserved LVEF [22]. However, the recent study showed that empagliflozin improved LV diastolic function only in patients HFrEF, but not in those with normal LVEF and without HF [23] (Table 1).

Recently published data from the IDDIA trial showed that dapagliflozin in addition to standard antihyperglycemic therapy in patients with type 2 DM was related with a significant improvement in LV diastolic dysfunction evaluated with diastolic stress echocardiography as compared with placebo [24]. The use of dapagliflozin resulted in a significant reduction of LV filling pressure evaluated by E/e′ during exercise in patients with type 2 DM.

Matsutani et al. reported that 3-month therapy with canagliflozin in DM patients (approximately 30% had CV disease) significantly reduced LV mass index and E/e′ [25]. Tofogliflozin was also proven to have positive effect on E/e′ in DM patients without CV disease who were treated with this agent for approximately 8 months [26] (Table 1).

### SGLT2 inhibitors and HFpEF

Data regarding use of SGLT2 inhibitors in HFpEF are scarce and mostly based on studies with limited number of participants. From pathophysiological perspective, the prescription of these medications would be justifiable because of favorable effect on LV filling pressure reduction and LV diastolic function improvement, which are the key points in treatment of HFpEF. In the recently published network meta-analysis, SGLT-2 inhibitors showed the largest risk reduction for HF hospitalization compared with placebo [7]. Additionally, SGLT-2 inhibitors were related with significant risk reduction in pairwise comparisons with both GLP-1R agonists and DPP-4 inhibitors. Study demonstrated 99.6% probability of SGLT-2 inhibitors being the optimal treatment for reducing the risk of HF outcome, followed by GLP-1 agonists (0.27%) and DPP-4 inhibitors (0.1%) [7].

Small investigation reported that 6-month therapy with dapagliflozin significantly improved LV diastolic function (E/e′ and left atrial volume index) in HFpEF patients with DM, even though there was no significant change in BNP [26]. Re-evaluation of these data demonstrated significant improvement not only in LV diastolic function but also in LV longitudinal strain only in HFpEF patients but not in HFrEF subjects [28]. This improvement in LV longitudinal strain was independently of demographic, clinical, and echocardiographic parameters associated with E/e′ after administration of dapagliflozin [28].

Another small study that investigated the effect of canagliflozin on LV remodeling in DM patients with HF (majority of patients—33/35 had HFpEF) showed that LV filling pressure, assessed by E/e′, significantly decreased after 6 months of treatment and effect was sustained even after 12 months of therapy [29] (Table 2).

**Table 2:** GLP-1R agonists and left ventricular diastolic function

Reference	Sample size	Medication	Follow-up period	Main findings
Bizino et al. [32]	49 DM patients	Liraglutide	26 weeks	Reduced early LV diastolic filling and LV filling pressure, as well as decreased LVEF, but it did not change cardiac output and cardiac index. LVEF remained within normal range
Saponaro et al. [33]	37 DM patients	Liraglutide	6 months	Significant improvement in LV diastolic function (E/A, E/e′)
Hiramatsu et al. [34]	139 DM patients	Liraglutide	48 months	E/e′ and LA volume index significantly improved
Ida et al. [35]	592 DM patients (meta-analysis)	Liraglutide	-	Liraglutide caused a significant improvement in LV diastolic function in comparison with other antidiabetic drugs (sitagliptin, linagliptin, pioglitazone, rosiglitazone, voglibose, and glimepiride)
Lambadiari et al. [36]	60 DM patients	Liraglutide	6 months	Improved arterial stiffness, LV myocardial strain, LV twisting and untwisting and NT-proBNP by reducing oxidative stress in patients with newly diagnosed DM, did not demonstrate significant improvement in LV diastolic function comparing with placebo
Kumarathurai et al. [37]	30 DM diabetes	Liraglutide	24 weeks	Liraglutide therapy did not improve any diastolic function parameters in subjects with DM, CAD, and preserved LVEF
Scalzo et al. [38]	23 DM patients	Exanatide	3 months	Improved significantly LV diastolic function and reduced arterial stiffness after 3 months of therapy, but did not improve functional exercise capacity
Zhang et al. [17]	4790 DM patients (meta-analysis)	Liraglutide Exanatide Albiglutide	-	GLP-1 agonists are more significantly associated with improved LVEF, LVESV and E/e′

*CAD* coronary artery disease *DM* diabetes mellitus *GLP-1* glucagon-like peptide-1 receptor *LA* left atrial *LVEF* left ventricular ejection fraction *LVESV* left ventricular end-systolic volume

The STADIA-HFpEF trial will evaluate the direct effects of 13-week therapy with dapagliflozin on LV stiffness in patients with HFpEF, and primary endpoint is echocardiographically derived change in E/e′, LV end-diastolic volume index, and change in mean LV e' [30].

The largest ongoing trial on this topic is the EMPEROR-Preserved study that enrolled 5988 symptomatic HF patients LVEF > 40% with and without type 2 DM of which one-third have LVEF between 40 and 50% (HF with mid-range LVEF), and two-third with LVEF > 50% (HFpEF) [8]. The presence of comorbidities such as diabetes (49%) and chronic kidney disease (50%) was common, and the majority of the patients are treated with angiotensin-converting enzyme inhibitors/angiotensin receptor blockers/angiotensin receptor-neprilysin inhibitors (80%) and beta-blockers (86%), and 37% of patients are on mineralocorticoid receptor antagonists [8]. The expected follow-up is 38 months. It is expected that this trial will provide many answers about the usefulness of SGLT2 inhibitors, particularly dapagliflozin, on outcome and LV remodeling in HFpEF and HFmrEF patients.

## The effects of GLP-1 receptor (GLP-1R) agonists on cardiovascular system

Secretion of GLP-1 stimulates insulin release by pancreatic beta-cells in a glucose-dependent correlation and reduces glucagon secretion by alpha-cells. GLP-1 reduces postprandial glucose by slowing gastric emptying, reducing intestinal glucose uptake, suppressing hepatic glucose production, and improving insulin sensitivity of muscle and liver [31]. The main limitation of endogenous GLP-1 is extremely short half-life (approximately 2 min) due to the activation of enzyme dipeptidylpeptidase-4 (DPP-4). There are only two possibilities to overcome this problem: inhibition of DPP-4 to prevent early breakdown of GLP-1 and invention of GLP-1R agonists that are resistant to degradation by DPP-4 and simulate the effect of GLP-1 [31].

Cardiovascular effects of GLP-1R are reduced activity of renin–angiotensin–aldosterone system, reduced oxidative stress, decreased blood pressure, improved endothelial function and microvascular perfusion, and reduced triglycerides and LDL levels. The negative effect might be increased sympathetic nervous system activity and direct sinoatrial node stimulation, which consequently increases heart rate [31].

### GLP-1R agonists and LV diastolic function

Growing body of evidence shows significant beneficial effect of GLP-1R agonists on LV diastolic function in patients with type 2 DM [32, 33]. Studies that investigated patients with type 2 DM and without cardiovascular disease revealed that liraglutide significantly increased *e*′ and decreased E/e′ and LV end-diastolic volume, which reflects reduction in LV filling pressure [32, 33] (Table 2). Hiramatsu et al. reported that liraglutide was effective for glucose and blood pressure reduction, reduced albuminuria, and improved LV diastolic function [34]. The authors showed that LV diastolic function was not improved by sitagliptin and linagliptin [34]. The meta-analysis that included 592 patients treated with different oral antidiabetic drugs (sitagliptin, linagliptin, pioglitazone, rosiglitazone, voglibose, and glimepiride) showed that only liraglutide was associated with significant improvement of LV diastolic function [35] (Table 2). Lambadiari et al. revealed that 6-month treatment with liraglutide improved arterial stiffness, LV myocardial strain, LV twisting, and untwisting by reducing oxidative stress in subjects with newly diagnosed DM [36].

Recent investigation that involved patients with DM and coronary artery disease with preserved LVEF treated with liraglutide did not demonstrate significant improvement in LV diastolic function comparing with placebo [37].

Administration of exanatide in patients with DM improved significantly LV diastolic function and reduced arterial stiffness after 3 months of therapy, but did not improve functional exercise capacity [38]. Zhang et al. in meta-analysis reported that GLP-1 agonists are significantly associated with improved LVEF, LV end-systolic volume, and E/e′ [17] (Table 2).

#### GLP-1R agonists and heart failure

GLP-1R agonists were not investigated in patients with HFpEF so far. However, studies conducted in DM patients with stable HFrEF did not support the use of liraglutide in patients with HFrEF and raised the questions about the safety of liraglutide in these subjects [39, 40]. The FIGHT study did not find significant changes in LVEF, pro-BNP, HbA1c, heart rate, LV end-systolic volume index, LV end-diastolic volume index, and 6-min walking when DM patients with HFrEF were treated with liraglutide for 6 months in comparison with placebo control group [39]. There were no significant differences in mortality or rehospitalization rate due to heart failure between liraglutide and placebo group [39]. The LIVE study revealed that liraglutide significantly reduced HbA1c and increased 6-min walking test [40]. However, it increased elevated heart rate and number of serious cardiac adverse events in comparison with control group. There were no statistical differences in LVEF, pro-BNP, LV end-systolic volume index, and LV end-diastolic volume index [40]. These findings raised some concerns with respect to the use of liraglutide in patients with chronic HFrEF. Even though dedicated studies were not positive, the recent meta-analysis reported 9% reduction in hospitalization rate for HF in patients treated with GLP-1R agonists [41].

Animal model of HFpEF showed that a 4-week GLP-1R agonist treatment via osmotic pumps significantly improved survival (70%) and reduced LV stiffness, LV diastolic dysfunction, and pulmonary congestion [42]. Another animal study in HFpEF mouse model reported that treatment with liraglutide attenuated the cardiometabolic dysregulation and improved cardiac function, with reduced cardiac hypertrophy, less myocardial fibrosis, and reduction of atrial weight, natriuretic peptide levels, and lung congestion [43]. These findings are encouraging and warrant further investigation in humans with HFpEF.

### DPP-4 and cardiovascular system

DPP-4 (dipeptidyl peptidase-4) inhibitors are responsible for the degradation of two gut-derived incretin hormones, GLP-1 and GIP (glucose-dependent insulinotropic polypeptide). DPP-4 inhibitors progressively replaced sulfonylureas in therapy of type 2 DM in many countries because they are not related with hypoglycemia or weight gain and they have good safety profile and comfortable usage even in patients with chronic renal failure [44]. Even though DPP-4 inhibitors mainly use GLP-1 pathway, they do not show that high level of cardiovascular protection as GLP-1R antagonists [44]. The reasons probably lay in the fact that some other DPP-4 regulated substrates are also cardioactive, such as stromal cell-derived factor-1 and brain natriuretic peptide [45], which is why controversies exist about the role of DPP-4 inhibitors in deterioration of HF and increased number of hospitalizations in these patients [44]. The majority of studies showed only non-inferiority of DPP-4 inhibitors with the respect of major adverse cardiovascular events (stroke, nonfatal myocardial infarction, and cardiovascular death) [46–48]. These are data from major trials that investigated the effect of saxagliptin, alogliptin, sitagliptin and linagliptin [45–47]. However, significant concern was raised because some studies showed increased rate of hospitalization due to heart failure [46]. Nevertheless, study that investigated the effects of sitagliptin in DM patients did not find any increase of hospitalizations due to HF [49]. This only confirms the ongoing controversies about the efficacy of DPP-4 inhibitors in DM patients with CV diseases.

### DPP-4 inhibitors and LV diastolic function

The limited data about the effect of DPP-4 inhibitors and LV diastolic function are inconsistent. Some studies showed the significant decrease in E/e′ ratio in poorly controlled DM patients treated with sitagliptin [50–52] (Table 3).

**Table 3:** DPP-4 inhibitoris and left ventricular diastolic function

Reference	Sample size	Medication	Follow-up period	Main findings
Nogueira et al. [50]	35 DM patients	Sitagliptin	24 weeks	Improvement in LV diastolic function was imporved in 75% of DM patients
Yamada et al. [51]	115 DM patients	Sitagliptin	24 months	Improvement of LV diastolic function (reduction in E/e′), but no change in LVEF
Leung et al. [52]	75 DM patients	Sitagliptin Vildagliptin Saxagliptin	12 months	Significant improvements in LV systolic, diastolic, and endothelial function
Kim et al. [53]	511,382 DM patients	Sitagliptin Linagliptin Vildagliptin Saxagliptin	12 months	The risk for HF was reduced in all of the patients, in patients with baseline cardiovascular disease, and in patients without baseline cardiovascular disease compared with patients for sulfonylurea-treated patients. Sitagliptin and linagliptin showed statistically lower risk for hospitalization for HF than for sulfonylurea
McMurrey et al. [54]	254 DM patients	Vildagliptin	52 weeks	No major effect on LVEF but increased LV volumes
Zhang et al. [17]	4790 DM patients (meta-analysis)	Sitagliptin Linagliptin Vildagliptin Teneligliptin Alogliptin Anagliptin	-	DPP-4 inhibitors are more strongly associated with a negative impact on LV end-diastolic volume

*DPP-4* dipeptidyl peptidase 4 *DM* diabetes mellitus *LV* left ventricle *LVEF* left ventricular ejection fraction

Small study that included 25 DM patients treated with different DPP-4 inhibitors (19 on sitagliptin, 5 on vildagliptin and 1 on saxagliptin) reported significant improvement in LV longitudinal strain and E/e′, surrogates of LV systolic and diastolic functions, and important improvement in endothelial function, after 12 months of treatment despite no significant differences in weight, blood pressure, or lipid parameters [52]. These effects provided some reassurance about the CV safety and efficacy of DDP-4 inhibitors (Table 3).

Meta-analysis that compared parameters of LV systolic and diastolic function in DM patients treated with different antidiabetic drugs showed no difference in all parameters of LV diastolic function (e′, E/e′ and E/A) in patients treated with DPP-4 comparing with those who were treated with competitors (thiazolidinediones, SGLT-2 inhibitors and sulfonylurea) [17] (Table 3).

### DPP-4 inhibitors and heart failure

Large investigation, which included more than 500,000 DM patients of whom half was treated with DDp-4 inhibitors, compared the effects of DPP-4 inhibitors with sulfonylurea and revealed no difference in the risk of HF between these two groups [53]. The authors even compared different DPP-4 inhibitors and found that sitagliptin and linagliptin were even associated with lower risk for hospitalization for HF than for sulfonylurea. Vildagliptin and saxagliptin also showed reduced risk for HF comparing with sulfonylurea, but the differences were not statistically significant [53].

A randomized placebo-control trial conducted in DM patients with HFrEF patients reported that vildagliptin did not change LVEF during 12-month therapy [54]. However, end-diastolic LV volume was significantly increased in patients treated with vildagliptin. The CARMELINA trial showed that linagliptin did not affect the incidence of hospitalization due to HF, the composite of cardiovascular death and hospitalization due to HF, or risk for recurrent HF events in comparison with placebo [55]. The meta-analysis that pooled data from five trials showed increased risk of admission for HF in patients treated with DPP-4 inhibitors versus control, but with borderline significance (HR 1.13; 95%CI 1.00–1.26) [56]. Nevertheless, it is difficult to make adjustment for all possible confounding factors in a meta-analysis, which can raise the question about its final results.

Other meta-analysis that included 4 trials, which investigated the effects of saxagliptin, alogliptin, sitagliptin, and linagliptin, separately analyzed the hospitalization rate due to HF in patients with and without prior history of HF, and found that DPP-4 inhibitors did not elevated risk of hospitalization due to HF in patients with previous HF, but in those DM patients without previous HF [57]. The same study reported borderline beneficial effect of GLP-1 receptor antagonists and significant positive effect of SGLT-2 inhibitors on the reduction of hospitalization in both groups, with and without history of HF. Conflicting results caused that authorities issued a warning about cautious prescription of DPP-4 inhibitors in patients with type 2 DM and a history of HF or kidney impairment [58]. The warning referred only on saxagliptin and alogliptin, but not other DPP-4 inhibitors. Nevertheless, one should underline that this is a very heterogeneous group of medications and some European Society for Heart Failure made clear discrepancy between saxagliptin that might increase risk of HF hospitalization and those DPP-4 inhibitors that have no impact on hospitalization (alogliptin, sitagliptin, vildagliptin, and linagliptin).

New studies that will be focused on this topic are warranted and particularly in patients with HFpEF in whom LVEF is not the main determinant. Ongoing TOPLEVEL study should determine the effect of DPP-4 inhibitor (teneligliptin) on LV diastolic function, using E/e′ as a main parameter, in type 2 DM patients and should answer the main question about the potential use of DPP-4 inhibitors in HFpEF [59]. Animal study reported that the inhibition of DPP-4 did not affect LV hypertrophy, but improved cardiac function and decreased myocardial and perivascular fibrosis [60]. These results indicate that DPP-4 inhibition decelerates the progression of HF by changing the quality and quantity of cardiac fibrosis, which might be the rational for usage of these medications in HFpEF patients.

## Conclusion

New antidiabetic drugs have incremental influence on DM treatment, but their influence on CV outcome and particularly the effects in HF patients are still largely uninvestigated and possibly underestimated. The available data are very encouraging about the use of SGLT2 inhibitors in HFrEF patients, but their role in HFpEF individuals remain to be investigated. The effects of GLP-1R agonists and DPP-4 inhibitors in HF patients are significantly less investigated and results are controversial, particularly in the later group of medications. Therefore, currently ongoing trials should provide more information about effects of these medications in patients with HF and particularly in those with HFpEF. Preclinical studies reported positive effects of new antidiabetic drugs in HFpEF, which is of a great clinical importance due to limited therapeutic options that are currently available in this large group of HF patients.
